# The Effect of Cognitive Impairment on Mortality Risk in Older Adults

**DOI:** 10.1093/geroni/igaf122.2569

**Published:** 2025-12-31

**Authors:** Delmarielinnette Castillo-Collazo, Héctor Gómez-Martínez, Amílcar Matos-Moreno

**Affiliations:** Albizu University, San Juan, Puerto Rico, United States; Albizu University, San Juan, Puerto Rico, United States; The Pennsylvania State University, University Park, Pennsylvania, United States

## Abstract

This study examined the influence of cognition on mortality among older adults in Puerto Rico, using data from the Puerto Rican Elderly: Health Conditions (PREHCO) study, collected between 2006 and 2022. Cognition has been consistently identified as a predictor of physical well-being in older adults, frequently influencing mortality. Although several studies have reported a relationship between cognitive decline and increased mortality risk in Caribbean populations, Puerto Rico has been excluded from these investigations. Given the rapid aging of this population and their high prevalence of chronic diseases, investigating how cognition influences survival outcomes among Puerto Rican older adults is essential. We analyzed longitudinal data from 2,289 participants (mean age = 74.12 years; 60.5% female) using a Cox regression model adjusted for sociodemographic and health-related covariates. Results indicated that higher cognitive scores were significantly associated with reduced mortality risk (HR = 0.945; CI: 0.924–0.967), reflecting a 5.5% decrease in risk with each incremental improvement in cognition. Additionally, advanced age, dependence in performing activities of daily living, and multiple chronic diseases significantly increased mortality risk. Men also exhibited greater mortality risk than women, while a higher level of education was associated with a reduction in mortality. Cognition emerged as a significant predictor of life expectancy among older Puerto Ricans, independently of other demographic and health factors. These findings highlight the importance of early detection of cognitive impairment and suggest the need for targeted interventions to enhance health outcomes in this population.

